# Toward Consensus on Correct Interpretation of Protein Binding in Plasma and Other Biological Matrices for COVID‐19 Therapeutic Development

**DOI:** 10.1002/cpt.2099

**Published:** 2020-11-21

**Authors:** Marta Boffito, David J. Back, Charles Flexner, Peter Sjö, Terrence F. Blaschke, Peter W. Horby, Dario Cattaneo, Edward P. Acosta, Peter Anderson, Andrew Owen

**Affiliations:** ^1^ Chelsea & Westminster Hospital London UK; ^2^ Department of Infectious Disease Imperial College London London UK; ^3^ Department of Pharmacology and Therapeutics University of Liverpool Liverpool UK; ^4^ Bloomberg School of Public Health Johns Hopkins University School of Medicine Baltimore Maryland USA; ^5^ Drugs for Neglected Diseases Initiative (DNDi) Geneva Switzerland; ^6^ Department of Medicine Stanford University School of Medicine Stanford California USA; ^7^ Centre for Tropical Medicine and Global Health Nuffield Department of Medicine University of Oxford Oxford UK; ^8^ Unit of Clinical Pharmacology ASST Fatebenefratelli Sacco University Hospital Milan Italy; ^9^ Department of Pharmacology and Toxicology University of Alabama at Birmingham Birmingham Alabama USA; ^10^ Skaggs School of Pharmacy and Pharmaceutical Sciences University of Colorado Aurora Colorado USA; ^11^ Centre of Excellence in Long‐acting Therapeutics (CELT) University of Liverpool UK

## Abstract

The urgent global public health need presented by severe acute respiratory syndrome‐coronavirus 2 (SARS‐CoV‐2) has brought scientists from diverse backgrounds together in an unprecedented international effort to rapidly identify interventions. There is a pressing need to apply clinical pharmacology principles and this has already been recognized by several other groups. However, one area that warrants additional specific consideration relates to plasma and tissue protein binding that broadly influences pharmacokinetics and pharmacodynamics. The principles of free drug theory have been forged and applied across drug development but are not currently being routinely applied for SARS‐CoV‐2 antiviral drugs. Consideration of protein binding is of critical importance to candidate selection but requires correct interpretation, in a drug‐specific manner, to avoid either underinterpretation or overinterpretation of its consequences. This paper represents a consensus from international researchers seeking to apply historical knowledge, which has underpinned highly successful antiviral drug development for other viruses, such as HIV and hepatitis C virus for decades.

The surge of cases during the coronavirus disease 2019 (COVID‐19) pandemic has led to the rapid implementation of clinical trials with drugs repurposed from existing antiviral or other drug classes. Some of these therapies have been used in the clinical setting with only limited *in vitro* data, and there is a danger in not applying the lessons learned from other viral infectious diseases in which successful interventions have been implemented. Currently, many ongoing trials have focused upon monotherapies that may provide insufficient drug exposures.^1^ Early HIV *in vitro* assay testing of antiretroviral compounds relied upon interpretation of *in vitro* effective concentration causing 50% of the maximal response (EC_50_) as a convenient measure for benchmarking clinical drug exposure. However, it is now accepted that plasma and/or compartmental antiviral drug concentrations need to remain above the protein‐binding adjusted EC 90% (EC_90_) or EC 95% (EC_95_) for HIV and need to remain so for the duration of their dosing interval in order to increase the chances of clinical benefit.^2^, ^3^ Whether this applies to the treatment of severe acute respiratory syndrome‐coronavirus 2 (SARS‐CoV‐2) is currently not known, but these same principles do apply to other viruses, such as hepatitis C virus, which has become the first virus that can be cured using small molecule drugs.

The importance of protein binding for antiretroviral drugs was recognized over 2 decades ago, and the field subsequently wrestled with the suitability of existing *in vitro* methodologies for rationalizing plasma pharmacokinetic efficacy cutoffs. For example, early studies with HIV protease inhibitors failed to demonstrate antiviral activity in trials despite plasma concentrations above the EC_90_ being achieved.^4^ The critical need for a consensus on standard procedures was recognized, and in June 2002, a panel of experts assembled in Washington, DC, to review and discuss the impact of plasma protein binding on the pharmacokinetics and activity of antiretroviral drugs.^3^ Many of the principles established at this meeting are of critical importance today while the international scientific community strives to bring forward options for treatment and prevention during the urgent unmet public health need presented by SARS‐CoV‐2. Several *ad hoc* and coordinated global screening programs have been initiated, but with few exceptions,^5^ emergent literature to date has not robustly integrated an understanding of protein binding into screening and development of drugs for SARS‐CoV‐2. Indeed, none of the studies cited in a recent review of *in vitro* data sought to determine protein‐adjusted activities using methods developed for other viruses.^1^ Revisiting the lessons learned over 2 decades ago in HIV is highly warranted.

## RELEVANCE OF *IN VITRO* PROTEIN BINDING INFORMATION TO THE INTERPRETATION OF EXPOSURE‐RESPONSE RELATIONSHIPS

In recent months, several papers have questioned the appropriateness of comparing *in vitro*‐derived activities to total plasma concentrations directly because only the unbound drug fraction is assumed to be able to exert antiviral activity.^6^, ^7^, ^8^ This phenomenon has been termed free drug theory (FDT),^9^, ^10^, ^11^ but it should be noted that not all drugs follow the principles of FDT. For example, drugs (or active metabolites) sometimes bind irreversibly to their target, resulting in a cumulative increase in irreversible binding to the target.^9^ Furthermore, drug transport proteins play an important role, which may also influence drug distribution resulting in the formation of sanctuary sites where viruses are able to replicate despite adequate systemic free drug concentrations.^12^, ^13^ Even for drugs that do obey the FDT, it is not appropriate to derive an unbound plasma concentration and use it to directly compare with *in vitro* antiviral activity for several reasons:

1. Drug binding *in vitro* is almost never zero because drugs bind to culture plastics and/or constituents of the culture media.^14^
2. The overwhelming majority of *in vitro* studies of drugs for treating SARS‐CoV‐2 to date have included protein in the culture media in the form of serum. The authors have reviewed preprints and papers that investigated the anti‐SARS‐CoV‐2 activity of 167 small molecule drugs. Across these papers, 88 reported use of 2% serum, 65 reported use of 10% serum, 11 reported use of 5% serum, 10 reported use of 2.5% serum, and 4 reported use of 12% serum.
3. Even small amounts of serum present in culture medium are capable of binding large amounts of drug. For example, a previous report indicated that a culture medium containing 5% serum was capable of binding 93.7% of lopinavir, rising to 96.1% at 10% serum, and 99.4% at 50% serum^15^ compared with 98–99% protein binding in human plasma.^16^ In line with this observation, by comparative equilibrium dialysis the maximal concentration (C_max_) of lopinavir in human plasma (15 μM) had the same amount of free (unbound to protein) lopinavir as 5 μM in cell culture medium containing 10% fetal bovine serum (FBS).^17^
4. Not all protein binding is the same. Albumin is considered to have a weak interaction with the drugs that it binds but is capable of associating with a large amount of drugs before saturation (low affinity/high capacity). Conversely, binding to alpha_1_‐AAG is considered to be high affinity/low capacity.^18^

## AN ILLUSTRATION OF THE CORRECT INTERPRETATION OF PROTEIN BINDING USING LOPINAVIR AND REMDESIVIR AS EXAMPLES

**Figure 1:**
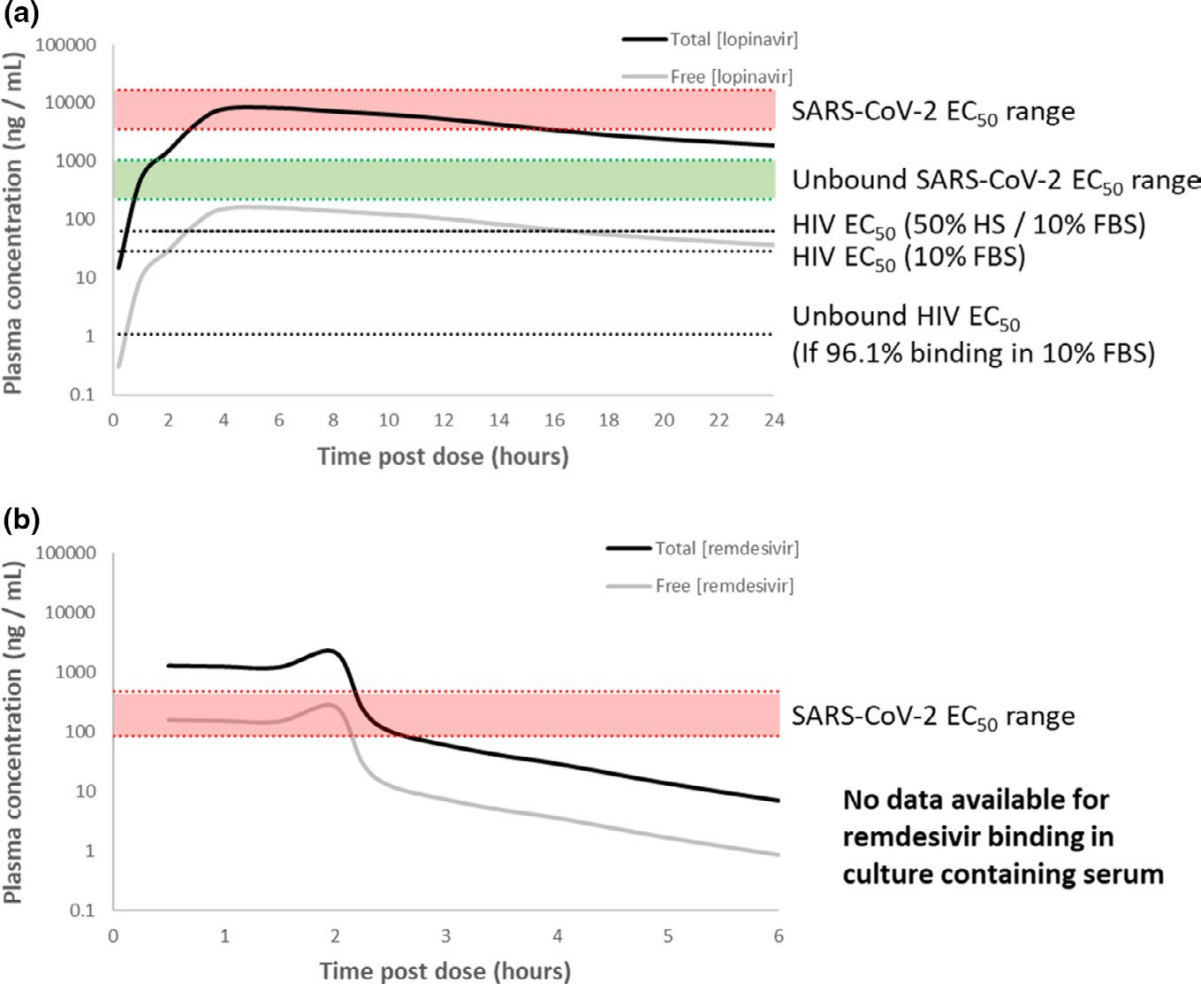
Comparison of human pharmacokinetics with *in vitro* derived anti‐severe acute respiratory syndrome‐coronavirus 2 (SARS‐CoV‐2) activities for lopinavir and remdesevir. For illustrative purposes, single dose data are presented for lopinavir (a) and remdesivir (b) because the need for rapid onset of anti‐SARS‐CoV‐2 activity may be needed and drugs like lopinavir take time to reach steady‐state pharmacokinetics. For remdesivir it should be noted that whereas this drug is given every 24 hours, it is cleared rapidly from the plasma and the published study only monitored plasma concentrations for 6 hours. Solid black lines represent published mean plasma concentrations whereas solid grey lines represent unbound drug concentrations derived from knowledge of the human plasma protein binding. The range of anti‐SARS‐CoV‐2 activities reported as effective concentration causing 50% of the maximal responses (EC_50s_) are shown by the shaded red areas. For lopinavir, where protein binding has been assessed in culture media containing serum, the derived unbound EC_50_ is shown by the green shaded area. The HIV EC_50_ values in the presence of human serum (HS) and/or fetal bovine serum (FBS) are also shown, along with an EC_50_ corrected for the expected free fraction in culture media. Further information and references to the source data are present in the main text.

Limited data are available to illustrate the importance of correct interpretation of plasma protein binding for SARS‐CoV‐2, because only remdesivir has been demonstrated to reduce the recovery time in a randomized controlled trial^19^ and the majority of publications across various small molecules only report *in vitro* EC_50_ values. However, the importance of understanding protein binding, using lopinavir/ritonavir and remdesivir as examples, is illustrated in **Figure** **1**. **Figure** **1a** shows the mean plasma concentration vs. time profile for 400 mg lopinavir after administration with 100 mg ritonavir to healthy male volunteers.^20^ However, it should be noted that lopinavir plasma concentrations in patients with COVID‐19 are higher than those in patients with HIV.^21^, ^22^, ^23^, ^24^, ^25^ The free drug fraction of lopinavir in human plasma is estimated to be ≤ 0.02 (i.e., ≤ 2%)^16^ and based upon this value the estimated free‐drug concentrations in plasma are also shown. The EC_50_ values of lopinavir against wild‐type HIV have been reported to be 28.3 ng/mL and 62.9 ng/mL in media containing 10% FBS or 50% human serum plus 10% FBS, respectively.^26^ Because 10% FBS has been reported to bind 96.1% of lopinavir,^15^ the free drug EC_50_ is estimated to be closer to 1.1 ng/mL. Hence, there is a high risk of misinterpreting the comparison of *in vitro* potency to *in vivo* efficacious concentrations because such comparisons need to be made based on the free concentrations both *in vivo* and *in vitro*. Equally important, the case for utility of lopinavir even in HIV is diminished if plasma free drug concentrations are compared directly with the *in vitro* activities that were themselves generated in the presence of serum.

Numerous *in vitro* anti‐SARS‐CoV‐2 EC_50_ values have been reported for lopinavir but for the purposes of illustration we have utilized 3,600 ng/mL with 5% FBS in VeroE6‐TMPRSS2 cells^27^ and 14,000 ng/mL with 10% FBS in Calu‐3 cells.^28^ Assuming 93.7% and 96.1% protein binding in media containing 5% and 10% FBS,^15^ the corresponding unbound EC_50s_ were derived as 226.8 and 532.2 ng/mL, respectively. Accordingly, the unbound plasma C_max_ (161.8 ng/mL) is between 22‐fold and 84‐fold lower than the *in vitro* EC_50_ and between 1.4‐fold and 3.3‐fold lower than the estimated free drug *in vitro* EC_50_. Importantly, this assessment of lopinavir pharmacokinetics does not robustly support antiviral activity across the entire dosing interval, whether total plasma concentrations are compared with EC_50_ or unbound plasma concentrations are compared with unbound EC_50_. The comparison of plasma pharmacokinetics to *in vitro* derived activity is highly sensitive to whether an EC_50_ or an EC_90_ is used and wide variability in values derived from different groups and different cell models is evident. Dramatic differences between EC_90_ and EC_50_ are also evident between different drugs or drug classes because of distinct differences in the slope of the concentration‐response curve, and it is never the case that an antiviral intervention seeks to inhibit replication by just 50%. Importantly, C_max_ exceeds EC_90_ in some but not all studies,^1^ but lowest concentrations of the drug before administration of the next dose (C_trough_) values do not exceed any of the currently reported *in vitro* activity measures irrespective of protein binding.

When considering other relevant tissue compartments for SARS‐CoV‐2, such as the central nervous system or lungs, the case for lopinavir activity is even less favorable. Several independent groups have estimated that the concentrations of lopinavir required to inhibit SARS‐CoV‐2 replication in epithelial lining fluid and cerebrospinal fluid may be several times higher than those measured *in vivo*.^7^, ^21^, ^29^ Thus, a consideration of free drug concentrations in other relevant matrices is likely to be needed to underpin successful therapeutic development, but a lack of standardized methodology complicated robust investigation.

**Figure****1b** shows the mean plasma concentration time profile for remdesivir following multiple dose administration of 150 mg to healthy volunteers.^30^ The free drug fraction of remdesivir in human plasma has been reported as 0.121 (12.1%)^31^ and this value has been used to derive the unbound plasma profile, which is also shown in **Figure** **1b**. The European Medicines Agency (EMA) compassionate use summary for remdesivir references two EC_50_ values as 0.137 μM (82.6 ng/mL) and 0.77 μM (464.0 ng/mL) and this range is also presented in **Figure** **1b**. This EC_50_ represents an extracellular metric and, therefore, it is appropriate to use plasma rather than intracellular concentrations in this comparison. However, as for other ProTide nucleoside prodrugs (e.g., sofosbuvir and tenofovir alafenamide), remdesivir accumulates intracellularly and its half‐life intracellularly is much longer than in plasma when determined in peripheral blood mononuclear cells from humans administered the drug intravenously.^32^ This sets ProTide nucleosides apart from drugs, such as lopinavir, that do not require intracellular bioactivation and for which the intracellular to plasma ratio remains constant across the dosing interval.^33^ Furthermore, drugs in this class are dependent upon multiple activation pathways that are reported to differ between *in vitro* and *in vivo* measures.^34^ Adequate plasma C_max_ is required to achieve target intracellular concentrations but maintaining plasma concentrations above *in vitro*‐defined extracellular cutoffs is not a prerequisite for success of this class because intracellular concentrations are maintained long after plasma concentrations fall below therapeutic concentrations. Thus, the presented comparison should be interpreted with caution for this class and robustly validated cell‐free assay systems will greatly aid understanding. Free drug concentrations of remdesivir in culture media containing serum or anti‐SARS‐CoV‐2 activity in the presence of different serum concentrations have also not yet been reported, and it is not possible to derive a prediction of the true unbound antiviral activity. The requirement for reliable demonstration of equivalency in rate and extent of intracellular prodrug bioactivation *in vitro* and *in vivo* makes a robust assessment highly ambitious. However, these data caution against the derivation of plasma unbound concentrations using a comparison with an antiviral activity measurement that was obtained in the presence of serum.

## EFFORTS TO BETTER INTERPRET PLASMA PROTEIN BINDING FOR APPLICATION IN HIV THERAPY

For HIV, the inhibitory quotient (IQ) was developed as a metric that combines plasma drug concentrations with the concentrations required to inhibit viral replication, to provide a better predictor of viral suppression. The IQ is derived by dividing the minimum plasma concentration (C_min_ or C_trough_) by an *in vitro* measure, such as EC_50_, EC_90_, or EC_95_. To correct for protein binding, serum‐free EC_50_ values were proposed, which would directly enable correction for protein binding^15^ and calculation of a more accurate IQ using plasma concentration data corrected for unbound fraction. However, human cell cultures require the presence of some serum to maintain viability and thus parallel experimental determination of activity and free drug measurement in varying amounts of serum was proposed to determine the free drug activity. The use of a serum protein binding correction factor used such an approach, and subsequently proved to be useful as a standardized approach for estimating the minimum drug exposure required for viral suppression.^2^ Although this is undoubtedly a more appropriate means of correcting for the effects of protein binding, it should be noted that concordance with minimum effective C_trough_ values were only observed for some antiretroviral drugs, whereas for other drugs this approach still under‐represented or over‐represented the targets.^2^

## OTHER IMPORTANT PHARMACOKINETIC CONSEQUENCES OF PROTEIN BINDING

Protein binding is an important parameter impacting several other pharmacokinetic considerations. Although plasma protein binding changes usually exert negligible effects on dose adjustment, with the exception of high clearance nonorally dosed drugs (e.g., intravenous), it may influence total clearance for low extracted drugs but not unbound clearance, and may or may not influence half‐life depending on the clearance and volume of distribution.^35^, ^36^ Various methods of assessment of free drug in plasma also differ in the values that they provide, and protein binding is different in different biological matrices (including tissue and intracellular compartments). Nonetheless, protein binding can be influenced by comorbidities (e.g., proteinuric kidney disease and liver impairment), differs in neonates, children, and pregnant women, and mediates some important drug‐drug interactions.^3^ AAG is an acute phase protein, which is induced during the systemic reaction to inflammation,^37^ and this may warrant particular consideration in the context of COVID‐19.^38^ The authors urge the scientific community to avail themselves of lessons learned in HIV and use them to apply a logical approach to interpretation of protein binding in the face of the new threat presented by SARS‐CoV‐2 infection.

## SUMMARY AND CONCLUSIONS

Understanding of the pharmacokinetics and pharmacodynamics of a drug in humans is a prerequisite for inclusion of regimens in clinical trials examining antiviral efficacy. Particular attention needs to be paid to *in vitro* inhibitory concentrations and ideally to using dosing regimens designed to achieve *in vivo* minimum effective concentrations in plasma (or intracellularly for nucleoside analogues like remdesivir or favipiravir). As the authors have emphasized above, care must be taken with interpretation of protein binding data where overly scrupulous application of *in vitro* data may discourage assessment of agents with therapeutic potential. Conversely, a lack of recognition of the impact of protein binding may promote evaluation of candidates that are not indicated. The current lack of an optimal pharmacodynamic parameter, such as quantitative viral load tests, has presented considerable difficulty in evaluating antiviral dosing regimens for SARS‐CoV‐2. Validation of pharmacokinetic/pharmacodynamic models will undoubtedly allow better prediction of activity, via concentration‐response curves and maximum effect (E_max_) models, to rapidly select drugs based upon efficacy. While an adequate surrogate marker of efficacy is being developed, it is critical that the clinical trial community better utilize available pharmacokinetic and *in vitro* activity data to make informed, evidence‐based selection of candidate therapies and dosing schedules. Lessons from past mistakes and *in vitro* model systems demonstrate that standardization and integrated empirical assessment of the impact of protein binding is required. No *in vitro* assay or prior knowledge of pharmacokinetics and pharmacodynamics can guarantee the success of a therapeutic agent, but if drugs do not achieve effective concentrations in relevant compartments, the chances of success in clinical trials are limited.

## Funding

The authors received no funding for the current work. P.S. acknowledges the Federal Ministry of Education and Research (BMBF) through KfW, Germany and thanks the donors contributing to DNDi’s overall mission: UK aid, UK; Médecins Sans Frontières International and the Swiss Agency for Development and Cooperation (SDC), Switzerland. P.H. acknowledges funding support from UKRI/NIHR (MC_PC_19056); Wellcome/DFiD (215091/Z/18/Z). P.A. acknowledges funding support from NIH (AI122298). A.O. acknowledges research funding from EPSRC (EP/R024804/1; EP/S012265/1), NIH (R01AI134091; R24AI118397), European Commission (761104) and Unitaid (project LONGEVITY).

## Conflicts of Interest

M.B. declares travel grants, speaker engagements, advisory roles, and research grants from Janssen, Roche, ViiV, Bristol‐Myers Squibb, Merck Sharp & Dohme, Gilead, Mylan, Cipla, and Teva. D.J.B. has received honoraria or advisory board payments from Abbvie, Gilead, ViiV, Merck, Janssen, and educational grants from Abbvie, Gilead, ViiV, Merck, Janssen, and Novartis. C.F. reports serving as a paid consultant for Gilead Sciences, Merck, and ViiV Healthcare. P.S. is an employee of Drugs for Neglected Disease initiative. D.C. has received consultant fees from ViiV Healthcare and speaker’s fees from MSD, Pfizer, Angelini, ViiV, and Janseen Cilag. P.A. has received personal fees and research funding paid to his institution from Gilead Sciences. A.O. is a Director of Tandem Nano Ltd. and has received research funding from ViiV, Merck, and Janssen, and consultancy from Gilead, ViiV, and Merck not related to the current paper. All other authors declared no competing interests for this work.
